# Do small samples bias the correlation between strength and jump performance? Multivariate insights into age and sex amidst strength saturation: an analysis of 1,544 participants from different sports

**DOI:** 10.5114/biolsport.2025.139858

**Published:** 2024-08-30

**Authors:** Michael Keiner, Konstantin Warneke, Andre Sander, Hagen Hartmann, Carl-Maximilian Wagner, Björn Kadlubowski, Andreas Wittke, Torsten Brauner, Andreas Konrad, David G. Behm, Klaus Wirth

**Affiliations:** 1 Department of Training and Exercise Science, German University of Health & Sport, Ismaning, Germany; 2 Department of Movement Sciences, University of Klagenfurt, Klagenfurt am Wörthersee, Austria; 3 German Luge and Bobsled Federation, Berchtesgaden, Germany; 4 Department of Sport Science, German University of Health & Sport, Ismaning, Germany; 5 Department of Biomechanics, German University of Health & Sport, Ismaning, Germany; 6 Institute of Human Movement Science, Sport and Health, University of Graz, Graz, Austria; 7 School of Human Kinetics and Recreation, Memorial University of Newfoundland, St. John’s, Canada; 8 Department of Training and Exercise Science, University of Applied Sciences Wiener Neustadt, Austria

**Keywords:** 1RM, Relative strength, Squat, Squat jump, Countermovement jump

## Abstract

Maximal strength is considered a fundamental aspect of athletic performance across a wide range of sports and is also needed for a range of activities of daily life. Yet, compared to males there are fewer publications examining females, with most showing similar coefficients of correlation between dynamic strength and different athletic performances. In both, males and females, results are biased by mostly small sample sizes (sample bias) leading to a fluctuation around the true correlation coefficient of the entire population. This crosssectional analysis involving 1544 participants employed multivariate and correlative analyses to clarify the importance of maximum strength in the parallel back squats on the jump performance controlling for variables such as type of sport, sex, age, and performance level. The analysis revealed two principal components that reflect distinct types of variability within the dataset: the first, primarily associated with performance capabilities, accounts for 58.45% of the variance, while the second, emphasizing demographic differences, accounts for a considerably lower variance of 25.08%. The correlation analyses in this study identified maximal strength as a significant factor influencing jumping performance, accounting for 48–53% of the variance in jump height. The analysis presents a saturation curve, with potential diminishing returns at higher strength levels. Age and sex had little to no effect on overall correlation coefficients. The overall correlation coefficients and the analyses for the subgroups (by sport and performance level) can differ considerably, which can be explained (mathematically) by the artificial formation of clusters, homogeneous subject groups, or small sample sizes.

## INTRODUCTION

Strength is considered a fundamental aspect of athletic performance across a wide range of sports and is also needed for a range of activities of daily life [1]. Maximum strength is significantly influenced by both morphological factors such as muscle mass and neural factors such as intramuscular and intermuscular coordination. Together with biomechanical factors, these elements determine the overall ability to generate force. Primarily, increases in streng = th capacity can be achieved through resistance training, which can enhance both morphological and neural conditions depending on the training methods employed. From a purely physical point of view, the execution of many explosive movements [2] such as sprinting and jumping can be highly influenced by the individual’s ability to exert high forces [3–10]. Force describes the mechanical interaction between bodies, which may be sufficient to change the state of motion of targeted objects (force = mass x acceleration) [11]. In sports, this principle can be frequently applied when force is exerted on an external object or resistance which is determined by muscle strength [12, 13]. Considering sport-specific requirements, the athlete may need to produce large forces against gravity when performing activities such as sprinting or jumping. Furthermore, in contact sports (e.g., football, wrestling) or individual sports (e.g., weightlifting, shot put) an external resistance or the opponent’s body mass is to be affected suggesting muscle strength is important. The constant in all these examples is that a limiting factor of muscular performance can be the muscular strength of the individual.

Accordingly, there is abundant evidence for positive correlations between isometric (r = |0.23–0.80|) [3, 7, 14, 15], isokinetic (r = |0.22–0.48|) [7], and free-weight squats (r = |0.28–0.94|) [3–5, 7, 9] and various sprinting and jumping performances, using absolute (r = |0.02–0.94|) [3, 5, 7, 9, 16–20] and relative strength (relative strength = maximum strength divided by body weight [REL]), r = |0.01–0.72|) [3, 5, 19, 20] values. Yet, compared to males there are fewer publications examining females, with most showing comparable coefficients between dynamic strength (one repetition maximum [1RM], REL) and sprinting or jumping with correlations ranging from r = |0.31–0.89| [18, 21, 22]. Independent of sex, results are biased by the mostly small study samples (sample bias) leading to a fluctuation around the true correlation coefficient of the entire population. Further study limitations that complicate the between-study comparison can be assumed to be high heterogeneity in the participants’ strength levels, sports, or ages [23]. Suchomel [24] hypothesized a non-linear relationship between back squat performance and speed-strength capability, like sprinting or jumping, since a diminished influence of maximal strength in high strength level athletes is expected [25], due to a saturation effect. Suchomel [24] categorizes REL into three different strength levels for the squat performance. The lowest strength level (0–0.5 REL-squat) is assumed to be limited by the individual’s motor learning ability and consequently underestimates actual strength performance, while, on the other hand, the authors described a limited transferability of strength to the target movement in high level athletes (> 2.0 REL-squat). Consequently, the best correlations with jumping performance are hypothesized to be in individuals, reaching 0.5–2.0 REL-squat. While this points to a generalized correlation, it is important to note that the magnitude of this relationship may vary depending on the training level of the subjects involved. This variability carries significant implications for training practices, suggesting that different adaptations may be necessary to optimize outcomes for different performance levels.

The objective of this study was to investigate the relationship between REL performance in parallel back squats and jumping performance (i.e., countermovement jumps [CMJ] and squat jumps [SJ]) across a large population, controlling for variables such as type of sport, sex, age, and performance level. Therefore, the generalizability of this correlation attempts to minimize sample bias to achieve high statistical significance. Furthermore, by identifying a potential saturation curve, this study brings important implications for training practices, emphasizing the importance of strength gains in enhancing jumping performance in different populations.

## MATERIALS AND METHODS

In accord with our study objective, data from 1544 (men: 1333; women: 211) participants were collected between 2014 and 2023.

During the COVID-19 pandemic, data collection had to be suspended, which accounts for the extended duration of the data gathering period. The data were collected in Germany at locations in and around Munich, Bielefeld, Frankfurt am Main, Hannover, and Hamburg. Testing was conducted by a six-member team (A.S., H.H., K.W., K.W., B.K., M.K.), with two testers always present on site during data collection for quality control purposes, recording the performances. Regular rater training sessions and quality control audits were held to ensure consistency and reliability in the data collection process. At each testing location, tests were carried out on 2 test days within a 1-week period. SJ and CMJ were tested on day 1, while 1RM was tested on the second day. For the evaluation, identical devices (contact mats [Refitronic, Schmitten, Germany]) were used. The mats were regularly replaced with identical ones due to wear and tear. The weights and the barbell were of the same type and were weighed before use as part of quality control. One week prior to test day 1, the participants completed a familiarization session for all tests on two separate days.

### Participants

Participants were sport students (men: 357; women: 163), athletes from team sports (soccer [men: 769; women: 9], water polo [men: 88, women: 0], football [men: 9, women: 0]; ice-hockey [men: 57, women: 0]), individual (youth, elite) sports (swimming [men: 13, women: 5], track cycling [men: 8; women: 4], ski jumping [men: 8; women: 0]) ski mountaineering [men: 10; women: 2] and cross-fit/powerlifting [men: 14, women: 28]). Participants’ training status was defined as recreationally active (sports students), performing 2–4 unsupervised training sessions per week (except recreationally power lifters / cross-fitter [performing 5–7 unsupervised sessions]), while athletes from soccer, football, ice-hockey swimming, track cycling, ski mountaineering, and ski jumping were categorized as highly trained athletes to elite athletes, performing 3–9 supervised sessions per week. The participants did not participate in fatiguing training sessions for a minimum of 2 days prior to testing. None of the participants reported any injuries at the time of testing.

Each participant or their parents (if the participant was younger than 18 years) were informed about the aims of the study and the experimental risks involved with the research and provided written informed consent. Furthermore, this study was performed in accordance with the Helsinki Declaration and was approved by the Universities Ethics Committee (DHGS-EK-202-002).

### Procedures

The warm-up for the jump tests consisted of non-specific running at low-to-medium intensity for approximately 5 minutes followed by coordination exercises, such as running with lifted knees, heel kicks, and side stepping, were performed for approximately 5 min. Subsequently, 3 acceleration runs over approximately 30 meters were performed with short walking breaks in between. The participants were tested for squat 1RM after 2–4 submaximal sets with 3–6 repetitions.

Jumping performance was evaluated using a contact mat (Refitronic, Schmitten, Germany), and the execution of each jump was visually monitored to ensure proper technique. The jump height was calculated from the flight time (gt^2^/8; with g being the gravitational acceleration (9.81 m · s^2^) and t being flight time in seconds). The squat jump was initiated at a knee angle of 90° without a countermovement. The countermovement jump was performed with a selfselected depth. Both jumps were performed without arm movement, the hands remained on the hips throughout the jump (akimbo). The participants performed 5 trials for each jump to achieve maximum jumping height. Between every jump, the athletes received a 1 min break. The test–retest reliability has previously been reported to be excellent with ICC = 0.97 [26].

Testing included the determination of the 1RM for a back squat (high bar) with the barbell positioned below the seventh cervical vertebra. The participants stood upright with a self-selected width of the feet, flexed their knees and hips to reach the parallel squat position with proper form (top of thigh breaking parallel) and returned to the starting position. Attempts failed when the trained spotter visually detected rounding of the back or insufficient squat depth. The determination of the 1RM was achieved within a maximum of 5 trials. The rest duration between attempts was at least 5 minutes. The test–retest reliability of 1RM for the squat is reported between ICC = 0.91–0.99 [27].

Absolute maximum strength values were divided by body mass to receive relative strength performance (REL-squat = 1RM/body mass). The strength classification from Suchomel [24] and others [28–35] was adopted in this study: strength level 1 (0.0–0.5 RELsquat), strength level 2 (> 0.5–1.0 REL-squat), strength level 3 (> 1.0–1.5 REL-squat), strength level 4 (> 1.5–2.0 REL-squat) and strength level 5 (> 2.0 REL-squat). Specific strength standards for young athletes (< 16 yrs.) are lacking [36]. Therefore, the younger athletes were also classified with the same strength levels 1–5.

### Statistical Analysis

The data was analysed using SPSS 26.0. (IBM, Ehningen, DE, Germany) to calculate descriptive statistics. In addition, analyses were conducted in R [37], Spearman’s Rho was calculated using the cor. test function and their respective confidence intervals with the package spearman.ci. The cluster analysis was carried out with the kmeans_res function and the Principal Component Analysis (PCA) with the pca_res function. Figures were produced using the package ggplot2 [38]. The significance level for all statistical tests was set at < 0.05.

The best performance for each test was used for the statistical analysis. Descriptive statistics for all measures are presented as mean ± standard deviation (SD). The statistical exploration was augmented by implementing PCA and k-means clustering to elucidate multivariate relationships within the dataset. The calculations revealed the absence of a multivariate normal distribution, which is not considered problematic [39]. PCA was utilized to reduce the data’s dimensionality, capturing variance related to strength and jump performance in the principal components. To determine the optimal number of clusters, an elbow analysis and a silhouette score were calculated, and a t-SNE plot was visually inspected by two independent raters (T.B. and M.K.). These methods collectively suggested that three clusters were most appropriate. Subsequently, k-means clustering identified distinct participant groups with similar characteristics. For detailed analysis the Kolmogorov-Smirnoff test for normality was calculated for total and sub-group’s data. The calculation by the Kolmogorov-Smirnoff test and the visual inspection of the histograms showed for the total group that data were not normally distributed. Since not all variables were normally distributed, bivariate one-tailed Spearman correlation analysis (Spearman rho) was used to assess the relationship between maximum strength with jump performance, respectively, for the total group and the subgroups. In addition, the partial correlation was calculated to control for a possible influence of the participants’ age. 95 percent confidence intervals (95%CI) were calculated for the correlation coefficients. Differences in correlation coefficients (after Fishers z-transformation) between subgroups (male vs. female) were tested according to Eid, Gollwitzer [40] with:

$$z=\frac{z_{1}-z_{2}}{\sqrt[{2}]{\frac{1}{N_{1}-3}+\frac{1}{N_{2}-3}}}$$

Effect sizes of differences (d) were calculated from the z-value with:

$$r=\frac{z}{\sqrt{n}}\text{and }d=\frac{2r}{\sqrt{1-r^{2}}}$$

In general, effect sizes defined as ^3^0.8 can be interpreted as large magnitude effects, ^3^0.5 can be interpreted as medium magnitude effects and ^3^0.2 can be interpreted as small magnitude effects [41].

## RESULTS

The descriptive statistics (means and standard deviations) of the anthropometric and performance data according to the strength level classification of strength classification from Suchomel [24] are presented in Tables 1 and 2, respectively.

**TABLE 1:** Means and standard deviations of age and anthropometric data.

Group	n	Age (yrs.)	Body mass (kg)	Height (m)	BMI
Level 1	65	14.5 ± 6.7 (10–38)	58.1 ± 22.9	1.61 ± 0.13	22.0 ± 7.3
Level 2	387	17.6 ± 5.8 (10–39)	62.7 ± 15.1	1.71 ± 0.11	21.3 ± 3.4
Level 3	803	19.3 ± 4.4 (10–38)	71.7 ± 12.3	1.77 ± 0.09	22.8 ± 3.0
Level 4	260	20.2 ± 4.4 (13–35)	70.5 ± 12.0	1.75 ± 0.08	22.8 ± 2.9
Level 5	29	27.8 ± 5.6 (16–40)	83.4 ± 9.8	1.75 ± 0.07	26.3 ± 3.5
Total	1544	19.0 ± 5.2 (10–40)	68.9 ± 14.4	1.74 ± 0.10	22.4 ± 3.5

yrs. = years, kg = kilogram, m = meter, Level = strength level, BMI: body mass index (kg/m^2^).

**TABLE 2:** Means and standard deviations of performance variables.

Group	Squat Jump (cm)	Countermovement Jump (cm)	Relative strength performance	Maximum strength performance (kg)
Level 1	22.4 ± 6.2	23.6 ± 5.9	0.34 ± 0.11	19.9 ± 9.8
Level 2	29.7 ± 5.5	31.7 ± 5.4	0.76 ± 0.14	48.3 ± 16.0
Level 3	35.8 ± 5.3	38.3 ± 5.6	1.25 ± 0.14	89.5 ± 18.1
Level 4	41.1 ± 5.6	44.1 ± 6.2	1.66 ± 0.13	116.7 ± 21.7
Level 5	45.3 ± 7.1	49.7 ± 7.2	2.16 ± 0.15	179.7 ± 25.5
Total	34.8 ± 7.2	37.2 ± 7.7	1.17 ± 0.39	82.5 ± 34.5

cm = centimeters, s = seconds, kg = kilogram, Level = strength level.

**FIG. 1:**
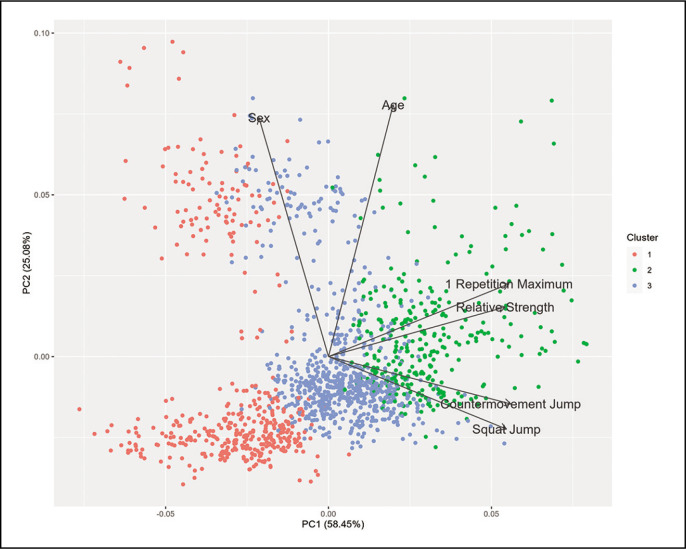
Cluster plot with loadings from Principal Component Analysis. *PC1 = Principal Component 1; PC2 = Principal Component 2*

**FIG. 2:**
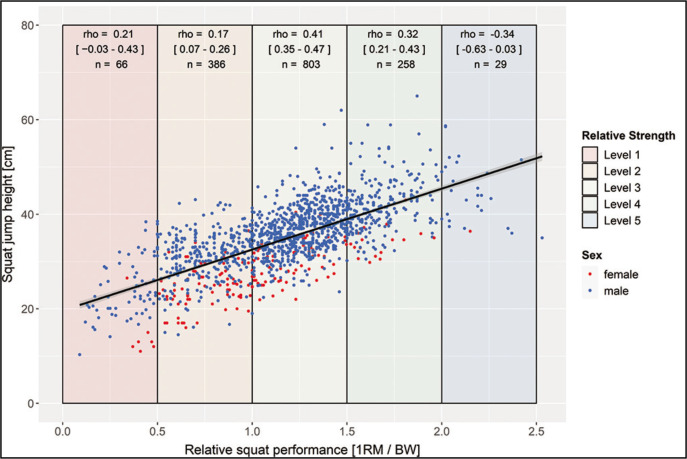
Scatterplot for relative squat and squat jump performance with trendline and 95% confidence interval (rho = 0.69 [p < 0.001; 95% CI = 0.66–0.72]; r_partial_ = 0.69 [p < 0.001; CI95% = 0.66–0.72]; n = 1544).

**FIG. 3:**
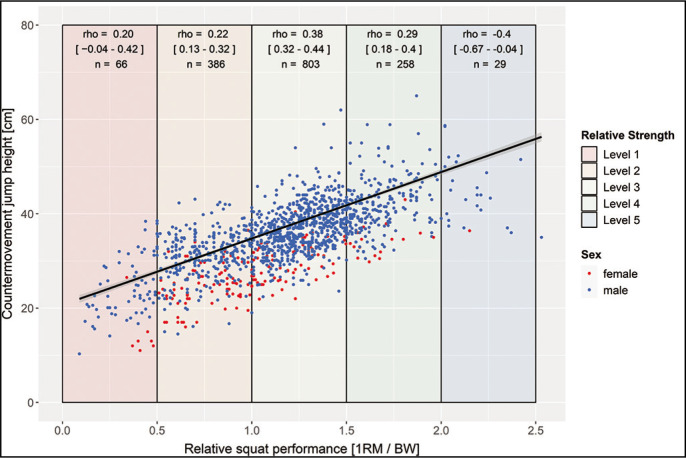
Scatterplot for relative squat and countermovement jump performance with trendline and 95% confidence interval (rho = 0.70 [p < 0.001; 95% CI = 0.68–0.73]; r_partial_ = 0.67 [p < 0.001; CI95% = 0.64–0.70]; n = 1544).

A silhouette score of 0.380 indicated a moderate level of separation between the clusters. This, in conjunction with the elbow analysis and visual inspection of the t-SNE plot, collectively suggested that partitioning the data into three clusters was the most appropriate, evidenced by an R-squared value of 0.556. The centroids of these clusters are detailed in Table 3, it is noteworthy that visual inspection of the cluster- (Figure 1) and scatterplots (Figure 2 and 3) does not reveal distinct cluster formations. Furthermore, it’s noteworthy that a visual analysis, which involved colouring the data points according to different sports, also failed to reveal any distinct patterns.

**TABLE 3:** Centroids of Clusters

Cluster	n	Age	Countermovement Jump	Squat Jump	Relative strength	Maximum strength	Sex
1	795	19.063	38.798	36.349	1.282	90.461	0.109
2	246	23.285	45.674	41.991	1.643	134.663	0.016
3	503	16.725	30.549	28.711	0.770	44.143	0.240

PCA played a pivotal role in understanding the data’s variance. The first principal component associated with performance capabilities, was responsible for about 58.45% of the variance, while the second emphasizing demographic differences accounted for approximately 25.08%. Together, these components captured over 83% of the data’s variability, highlighting their significance in the dataset. Notably, the loading scores for the first dimension ranged between 0.893 to 0.918 for the variables related to jumping and strength performances, with age contributing a score of 0.336 and sex showing a score of -0.434. The second dimension was primarily influenced by age, with a loading score of 0.835, and sex, which had a loading of 0.799.

Following the normal distribution of performances, only a few participants are found in each of the extreme strength levels 1 and 5 (Figure 2 and 3). Considering the whole sample participants with higher strength levels exhibit higher mean values in jump performance. Over the total group, REL-squat (rho = |0.69–0.70|) and 1RM (rho = |0.70–0.73|) correlate with performance parameters significantly (p < 0.001). Strength variables explain 48–53% of the variances of the tested jumping performances. No significant differences were found in the correlation coefficients between jumping performances and absolute or relative strength performances. Controlling for a possible age effect by partial correlation modifies the coefficients only marginally (rho = |0.67–0.73|). The correlation analysis of the various strength level subgroups revealed lower correlations within the subgroups (strength levels). Only the sub-group of strength level 1 showed no significant (p < 0.05) correlation between strength and jump performance parameters. A significant negative correlation was calculated in the strength level 5.

Correlation analysis by sex showed significant (p < 0.001) correlation coefficients between strength and jump variables (rho = 0.63–73) for all analysed variables (Table 4). A significant difference between the coefficients of both sexes could only be calculated between 1RM and CMJ. However, the effect size difference is trivial (d = 0.11). Table 5 shows the calculated correlation coefficients by sport. A difference calculation of the coefficients was omitted due to the extremely different numbers of cases.

**TABLE 4:** Spearman correlation coefficient, 95 percent confidence intervals and differences of correlation coefficient for subgroups (sex)

		Male	Female	Correlation coefficient differences
REL-squat	SJ	0.69 (0.66–0.72)	0.73 (0.67–0.79)	n.s. z = 1.24; p = 0.21; d = 0.06)
	CMJ	0.70 (0.67–0.73)	0.69 (0.62–0.76)	n.s. z = 0.13; p = 0.90; d = 0.01)

1RM	SJ	0.67 (0.64–0.70)	0.65 (0.57–0.73)	n.s. z = 0.43; p = 0.67; d = 0.02)
	CMJ	0.72 (0.69–0.75)	0.63 (0.55–0.71)	sig. z = 2.11; p = 0.04; d = 0.11)

REL-squat = relative strength performance; 1RM = one repetition maximum; SJ = squat jump, CMJ = countermovement jump, n.s. = not significant, sig. = significant

**TABLE 5:** Spearman correlation coefficient and 95 percent confidence intervals for subgroups (sport).

Subjects	n	REL-squat		1RM	

		SJ	CMJ	SJ	CMJ
Soccer	778 (m:769; w:9)	0.63* (0.59–0.67)	0.64* (0.60–0.68)	0.64* (0.60–0.68)	0.67* (0.63–0.71)
Waterpolo	88 (m:88; w:0)	0.67* (0.55–0.79)	0.70* (0.59–0.81)	0.69* (0.58–0.80)	0.71* (0.61–0.82)
Ice-Hockey	57 (m:57; w:0)	0.20 (-0.06–0.46)	0.23* (-0.02–0.48)	0.31* (0.07–0.55)	0.32* (0.08–0.56)
Football	9 (m:9, w:0)	0.64* (0.17–1.0)	0.60* (0.09–1.0)	-0.27 (-1.0–0.47)	-0.17 (-0.95–0.61)
Sport Students	520 (m:357; w:163)	0.71* (0.67–0.75)	0.69* (0.65–0.74)	0.77* (0.74–0.81)	0.76* (0.72–0.80)
Cross-Fit / Powerlifter	42 (m: 14, w:28)	0.86* (0.78–0.94)	0.85* (0.76–0.94)	0.76* (0.63–0.89)	0.75* (0.28–0.75)
Swimming	18 (m:13, w:5)	0.70* (0.44–0.96)	0.67* (0.39–0.95)	0.71* (0.46–0.96)	0.66* (0.61–0.89)
Track Cycling	12 (m:8; w:4)	0.42 (-0.12–0.96)	0.57* (0.13–1.0)	0.49 (-0.01–0.99)	0.68* (0.33–1.0)
Ski Mountain-eering	12 (m:10; w:2)	0.43 (-0.10–0.96)	0.22 (-0.40–0.84)	0.67* (0.31–1.0)	0.39 (-0.16–0.94)
Ski Jumping	8 (m:8; w:0)	0.38 (-0.37–1.0)	0.43 (-0.29–1.0)	0.37 (-0.39–1.0)	0.41 (-0.32–1.0)

REL-squat = relative strength performance; 1RM = one repetition maximum; SJ = squat jump, CMJ = countermovement jump,

* = significant (p < 0.05).

## DISCUSSION

This study, with its extensive sample of over 1500 participants, provides a nuanced understanding of the relationship between maximal strength in parallel back squats and jumping performance, factoring in age, sex, and training level. Notably, despite the thorough analysis, the dataset revealed no discernible clustering patterns, even when segmented by variables. The multivariate analyses indicate two principal components that reflect distinct types of variability within the dataset: the first, primarily associated with performance capabilities, accounted for 58.45% of the variance, while the second, emphasizing demographic differences, accounted for a considerably lower variance of 25.08%. The correlation analyses in this study identified maximal strength as a significant influencing factor on jumping performance, accounting for 48–53% of the variance in jump height in the overall statistics. Concurrently, the employed moderators – age, sex, and training level – can be attributed to variations in the results across different subgroups.

In accordance with the literature, reported correlation coefficients (rho = |0.69–0.73|) can be considered the approximate means of the range of correlations for free-weight squats (r = |0.02–0.94|) [3–5, 7, 9]. In principle, this does not seem surprising, considering smaller samples used in the referred studies led to a higher sample error compared to the present study. It could not be confirmed in this study that strength relative to body mass could be considered a better predictor of explosive strength performance than absolute strength in general [42]. Both absolute and relative strength show similar explained variance (48%–53%) underlining on the one hand, the relevance of maximum strength on explosive- and speed strength, on the other hand, showing the relevance of other performance parameters (e.g., technique) to influence jumping performance.

Indeed, lower subgroup correlation coefficients were observed compared to the total group, which can be explained mathematically by cluster forming within a subgroup, considering smaller samples from specialized cohorts causing a higher dispersion around the true coefficient. Furthermore, topic specific approaches can be speculated to impact the relationship between maximal and speed strength performance. Considering the limitations of strength transferability in the low trained group as well as the high trained group, the present results are in line with theories pointing out limited validity of performance testing (maximum strength and jump) in untrained participants due to technique problems. Indeed, no significant correlation was obtained for the untrained group (level 1). Even though very high strength values overall were associated with high jump performance (level 5), increased dispersion around the trend line started at level 4. In the high trained group (level 5), there was a negative correlation, indicating a limited transferability of maximum squat strength to explosive and speed strength. These controversial results could also be attributed to the smaller number of participants underlining the hypothesized saturation of the regression curve. However, the assumption is based on 3 out of 4 of the strongest athletes (powerlifters, see figure 2 and 3). In powerlifters, a significant proportion of the 1RM may derived from the back (technique) which could affect the correlation with jump performance, which primarily relies on leg strength. In contrast, it could be hypothesized that other factors, such as explosive strength/rate of force development should also be considered in trained (REL > 2.0) participants [43]. Although no underlying physiological parameters were measured within this study, it is well known that either maximal strength as well as speed strength/explosive strength performance requires morphological (e.g., high muscle mass) and neural basis (e.g., maximal motor unit recruitment and firing frequency, synchronization), supporting the results for the level 2–4 groups [44]. Therefore, an athlete with a high REL does not necessarily exhibit a high rate of force development. Consequently, Hill [45] demonstrated, with increasing muscle contraction speed, the achievable force height decreases, suggesting that strength becomes less utilizable as movement speed improves. Furthermore, jumping performance may also be subject to saturation, as the faster execution of movements (indicative of higher jumping performance) places greater demands on coordination, which could serve as a limiting factor [46–49].

The data from this study indicate no to moderate influence of the variables type of sport, age, and sex on the level of correlation between maximum strength and jumping performance. In the multivariate analysis, the variables of sex and age were included, while the variable of the type of sport was excluded due to its scale level. When considering the coefficients by sport, only the sports with a low number of participants (e.g., football, track bike, and ski jumping) show partly deviating (lower) coefficients suggesting larger sampling errors based on homogeneous performance (leading to lower variance, thus to lower coefficients). However, based on the available data, no subgroup analysis for sports could be performed. That specialized (strength/sport) training strategies can lead to skewing the relationship is conceivable, but this explanatory approach remains speculative. In the analysis’s first principal component, age demonstrated a moderately low positive loading (0.336), suggesting a moderate impact on athletic performance and indicating potential differences in characteristics between older and younger participants. Maturity and fewer years of training can be posited as potential explanatory factors for the moderate positive loading of age on the first principal component. The analysis of sex, with a loading of -0.434, reveals distinct performance patterns, though this observation may be attributed to the sample’s imbalance. In the second principal component, both age (0.835) and sex (0.799) showed significantly high loadings, indicating that this component is strongly influenced by age and sex differences. Therefore, the second principal component likely captures variations among participants related to demographic factors such as age and sex, contrasting with the first principal component’s focus on pure performance traits. Consequently, controlling coefficients of correlation analysis for sex, the data of this study only show a significant difference in one calculation (CMJ vs. 1RM), although this can be considered trivial (d = 0.11). This is analogous to the overall data literature conformity. Thus, sex may not be assumed to be an influencing factor of the strength and power relationship. Calculating the partial correlation (controlling for age) changed the coefficients only marginally (r = 0.69 vs. r = 0.69 and r = 0.70 vs. r = 0.67). Therefore, the data of this study strengthen that maximum strength, as a basic strength ability, positively influences the performance of explosive-strength.

No study is without its limitations, and this one is no exception. Among the constraints to consider in this study, that unbalanced samples could limit the interpretability of the results. Even though a comparatively high number of 211 female participants was included, with 1333 males, there were substantially fewer female participants included in this study. This is generally a problem in the literature [50], although this study includes a comparably high number of female participants associated with the unequal number of participants from selected sports (ad-hoc samples). This complicates a comparison between the sports. It should also be noted that the extreme strength levels (1 and 5) have the lowest number of participants, which can be explained by the normal distribution of performances. A further increase in the number of participants would probably not change the distribution between the strength levels.

Despite its limitations, this study provides important insights for training practices: 1) Up to a relative strength level (REL) of 2.0, high levels of maximum strength correlate with high levels of jump performance. Therefore, enhancing maximum strength to improve power performance is recommended. 2) Since the strength requirements of a sport follow an optimal trend, it is crucial to consider the cost-benefit aspects when evaluating exceptionally high strength values (REL > 2.0). The training volume required for further strength increases should be critically assessed to determine whether the benefits justify the effort. 3) Higher maximal strength, regardless of the sport, leads to better jumping performance. If high power output, such as jumping, is a crucial component of the sport’s performance requirements, then an increase in strength is strongly recommended. 4) Since for both sexes and all age groups the positive association could be confirmed, this study underlines the importance of maximal strength development in all age group as well as in male and female athletes, while calling for long-term research of resistance training to confirm a causal relationship via longitudinal study designs.

## CONCLUSIONS

In summary, the multivariate analysis indicates that age and sex minimally impact the relationship between maximal strength and jump performance, suggesting a general applicability across demographics [2]. The relationship between strength and jump performance presents a saturation curve, with potential diminishing returns (plateau effect) at higher strength levels. This study also highlights the impact of sampling errors on the variability of correlation coefficients, emphasizing the significance of adequate sample sizes for reliable results. Individuals with REL < 2.0 in the squat appear to benefit especially from an increase in maximum strength. It is also important to consider the increasing training effort required to achieve very high levels of strength (REL > 2.0). Even though further improvements in strength might be fundamentally beneficial, there comes a point where the investment in training may not proportionally correspond to the gains in performance. This imbalance between effort and reward needs to be carefully weighed, especially at advanced levels of strength training (REL > 2.0). Longitudinal studies have already shown that strength training is preventive, safe, and performance-enhancing [31, 51, 52]. From this, it can be concluded that long-term strength training is recommended for persons (independent of sex, age, sport, or strength training level) to increase performance in jumping, and thereby, to potentially increase their game performances. However, a high correlation between strength and jump performance is more likely to be achieved with a high level of strength that is optimal for the sport or activity (training specificity) [53]. Future investigations should address whether athletes with very high strength levels profit from further increasing their non-sport specific maximal strength or whether that training time could be spent more wisely otherwise (costs vs. benefit).
